# 4540 THE IMPACT OF SURGEON AND HOSPITAL VOLUME ON 30-DAY OUTCOMES AND COST FOR RENAL CANCER SURGERY

**DOI:** 10.1017/cts.2020.436

**Published:** 2020-07-29

**Authors:** Julia Wainger, Joseph Cheaib, Hiten Patel, Mitchell Huang, Michael Biles, Michael Johnson, Joseph Canner, Mohamad Allaf, Phillip Pierorazio

**Affiliations:** 1 Johns Hopkins University School of Medicine

## Abstract

OBJECTIVES/GOALS: Provider and hospital factors influence quality, but granular data is lacking to assess their impact on renal cancer surgery. The Maryland Health Service Cost Review Commission (HSCRC) is an independent state agency that promotes cost containment, access to care and accountability. Within HSCRC, we aimed to assess the impact of surgeon and hospital volume on 30-day outcomes after renal cancer surgery. METHODS/STUDY POPULATION: Data on renal surgery were abstracted from the Maryland HSCRC from 2000-2018. We excluded patients younger than 18, patients without a diagnosis of renal cancer, and patients concurrently receiving another major surgery. Volume categories were derived from the distribution of mean cases performed per year. We used adjusted multivariable logistic and linear regression models to identify associations of surgeon and hospital volume with the length of stay, days in intensive care, cost, 30-day mortality, readmission, and complications. RESULTS/ANTICIPATED RESULTS: A total of 10,590 surgeries, completed by 669 surgeons at 48 hospitals, met criteria. The 25^th^ percentile for cases per year was 1, the 50^th^ percentile was 1.2, and the 75^th^ percentile was 2.6. After adjusting for patient factors and cumulative surgeon experience, high volume surgeons had the greatest decrease in length of stay (β: −1.65, P<0.001) and mortality risk (OR: 0.27, 95% CI: 0.10-0.71) compared to rare volume surgeons. Low volume surgeons had the greatest cost decrease (β: -$7,300, P<0.001) compared to rare volume surgeons. Medium volume hospitals had statistically lower average costs than rare volume hospitals (β: $−2,862, P = 0.005). There were no other clinically and statistically significant relationships between volume and measured outcomes. DISCUSSION/SIGNIFICANCE OF IMPACT: Almost half of the urologists studied performed an average of one renal cancer case per year. Greater surgeon volume was associated with shorter length of stay and decreased mortality risk. Hospital volume did not have a meaningful relationship to outcomes. Other factors such as tumor, surgeon, and hospital characteristics or case-mix may associate with outcomes and could be confounders.

